# A new lightweight network for efficient UAV object detection

**DOI:** 10.1038/s41598-024-64232-z

**Published:** 2024-06-10

**Authors:** Wei Hua, Qili Chen, Wenbai Chen

**Affiliations:** Beijing Information Science and Technology University, Beijing, 100192 China

**Keywords:** Lightweight network, Object detection, Convolutional neural network, Unmanned aerial vehicle (UAV), Computer science, Information technology

## Abstract

Optimizing the structure of deep neural networks is essential in many applications. Especially in the object detection tasks of Unmanned Aerial Vehicles. Due to the constraints of the onboard platform, a more efficient network is required to meet practical demands. Nevertheless, existing lightweight detection networks exhibit excessive redundant computations and may yield in a certain level of accuracy loss. To address these issues, this paper proposes a new lightweight network structure named Cross-Stage Partially Deformable Network (CSPDNet). The initial proposal consists of a Deformable Separable Convolution Block (DSCBlock), separating feature channels, greatly reducing the computational load of convolution, and applying adaptive sampling to the separated feature map. Subsequently, to establish information interaction between feature layers, a channel weighting module is proposed. This module calculates weights for the separated feature map, facilitating information exchange across channels and resolutions. Moreover, it compensates for the effect of point-wise (1 $\times$ 1) convolutions, filtering out more important feature information. Furthermore, a new CSPDBlock is designed, primarily composed of DSCBlock, establishing multidimensional feature correlations for each separated feature layer. This approach improves the ability to capture critical feature information and reconstruct gradient paths, thereby preserving detection accuracy. The proposed technology achieves a balance between model parameter size and detection accuracy. Furthermore, experimental results on object detection datasets demonstrate that our designed network, using fewer parameters, achieves competitive detection performance results compared to existing lightweight networks YOLOv5n, YOLOv6n, YOLOv8n, NanoDet and PP-PicoDet. The optimization effect of the designed CSPDBlock, using the VisDrone dataset, is validated when incorporated into advanced detection algorithms YOLOv5m, PPYOLOEm, YOLOv7, RTMDetm and YOLOv8m. In more detail, by incorporating the designed modules it was achieved that the parameters were reduced by 10–20% while almost maintaining detection accuracy.

## Introduction

Unmanned Aerial Vehicle (UAV) perform object detection tasks using onboard cameras and embedded systems. They also find extensive research and applications in civilian and military domains, including UAV reconnaissance^1^, traffic monitoring^2^, and personnel rescue^3^. Moreover, Deep Learning (DL) technology systematizes the identification and localization of objects within images. This feature enhances the perceptual functions of UAV, particularly in a scenarios with limited human-drone interaction, providing fundamental technical support for autonomous detection and flight. To improve the feature quality of small targets for drones, SmallTrack^4^ uses a small wave pool layer to avoid losing small target information during downsampling and highlights the classification response of small objects through a graph enhancement module, which improves detection accuracy but is difficult to meet real-time requirements. MobileTrack^5^ proposes a lightweight convolutional network and enhances the feature information of targets by designing an efficient target perception module for local cross-channel information exchange, achieving a balance between speed and accuracy.

Convolutional neural networks (CNNs) have been increasingly utilized in the field of object recognition and have achieved significant advancements. Specifically, You Only Look Once (YOLO) series^6–10^ might be the most popular detectors in practical applications due to their well-balanced detection accuracy and speed. However, the inference of these detectors still demands high-performance computing and significant runtime memory usage to uphold good detection performance.

As the demand for detection performance increases, so does the parameter count of networks, leading to efficiency concerns in network design. Efficiency primarily revolves around balancing the model’s storage, computational requirements, and performance. Initially, high-performance networks typically encompass several weight parameters, demanding substantial memory resources for storing these parameters. Moreover, real-time requirements in practical scenarios are pressing, necessitating either enhanced processor capabilities or reduced computational load of the network to achieve this goal.

Thus, reducing the model scale and computational complexity while preserving accuracy without compromise has become a crucial research focus. Despite the development of several efficient lightweight methods, such as model pruning^11^ and knowledge distillation^12^, they primarily optimize the post-training models of the designated network.For instance, in 2016, Google team introduced the lightweight networks concept^13^, allowing direct training of Deep Neural Networks (DNNs) tailored for edge device deployment from the network structure itself.

In recent works focusing on UAV embedded deployment^14,15^, LWUAVDet^16^ enhances multi-scale representation at each stage by designing a topology structure that should be expanded and refined in the neck layer, and designs a pixel encoder-decoder for spatial and channel dimension exchange to achieve flexible and effective head feature extraction. GCL-YOLO^17^ proposes a lightweight YOLO network based on Ghost Conv, reducing the parameter quantity by establishing a Ghost Conv-based backbone network with fewer parameters. UAV-Net^18^ introduces a new filtering pruning method, which automatically compresses the trained network iteratively. LODNU^19^ reconstructs the backbone network with depthwise separable convolution to reduce model parameters and embeds improved coordinate attention points in the backbone network to enhance the extraction capability of key target features. Meanwhile, an adaptive scale-weighted feature fusion module is incorporated into the path aggregation network to improve the accuracy of multi-scale object detection and achieve a balance between model size and accuracy.

To simplify complex model structures, researchers have developed some lightweight modules for network design. For instance, they have proposed the Fire module in SqueezeNet^20^, the Depth-wise Separable Convolution in MobileNet^13^, and modules consisting of channel shuffle and group convolution in ShuffleNet^21^. Several cutting-edge lightweight networks^22–27^ leverage depth-wise separable convolution or group convolution to decrease network computation. However, since these operations don’t alter the feature channels’ count, integrating information across channels and adjusting them to a specific dimension necessitates 1$\times$1 convolutions. Consequently, the use of such convolution in lightweight networks consumes substantial computational resources, resulting in constrained channels and a moderate decline in model accuracy. Therefore, the Inverted Residual Block (IRB), proposed by MobileNetv2^28^, can mitigate feature degradation and enhance representational capabilities. Apart from IRB, several efficient basic blocks^23,29,30^ have been designed to improve the representational capabilities of lightweight CNNs. However, inconsistent architectures could lead to redundant structures, emphasizing the importance of the efficient structural design for both the lightweight nature and performance impact of networks.

In the DNNs design process, many studies have proven that increasing the network depth is an effective means to improve the system’s accuracy^31–33^. However, as the network depth increases, obstacles, such as gradient explosions, hinder convergence during training. Furthermore, when deep networks start converging, the accuracy tends to saturate and then rapidly degrades. Additionally, directly expanding Neural Networks (NNs) often brings more parameters and computational burdens, hampering the deployment of advanced technologies on edge devices. Several researchers seek more efficient network structural designs to address these issues^25,34–36^. For instance, ResNet^31^ addresses this issue by providing a form of residual connectivity and build, an efficient network structure based on this design concept. Unlike aggregated features of ResNet, DenseNet^36^ connects each layer to all other layers in a feed-forward fashion, leading to modify the gradient vanishing problem, enhance feature propagation, encourage feature reuse, and big decrease the number of parameters. However, this densely connected computation leads to a quadratic increase in memory access overhead with network’s depth, resulting in complex computational and higher energy consumption. To address this challenge, CondenseNet^37^ proposed a novel network architecture relying on learnable packet convolution. This architecture automatically streamlines the network during training, identifies the optimal input-output connection pattern, and transforms it into a standard packet convolution structure in the end. However, existing CNNs, when lightened, effective scale down model parameters but often sacrifice accuracy.

**Figure 1:**
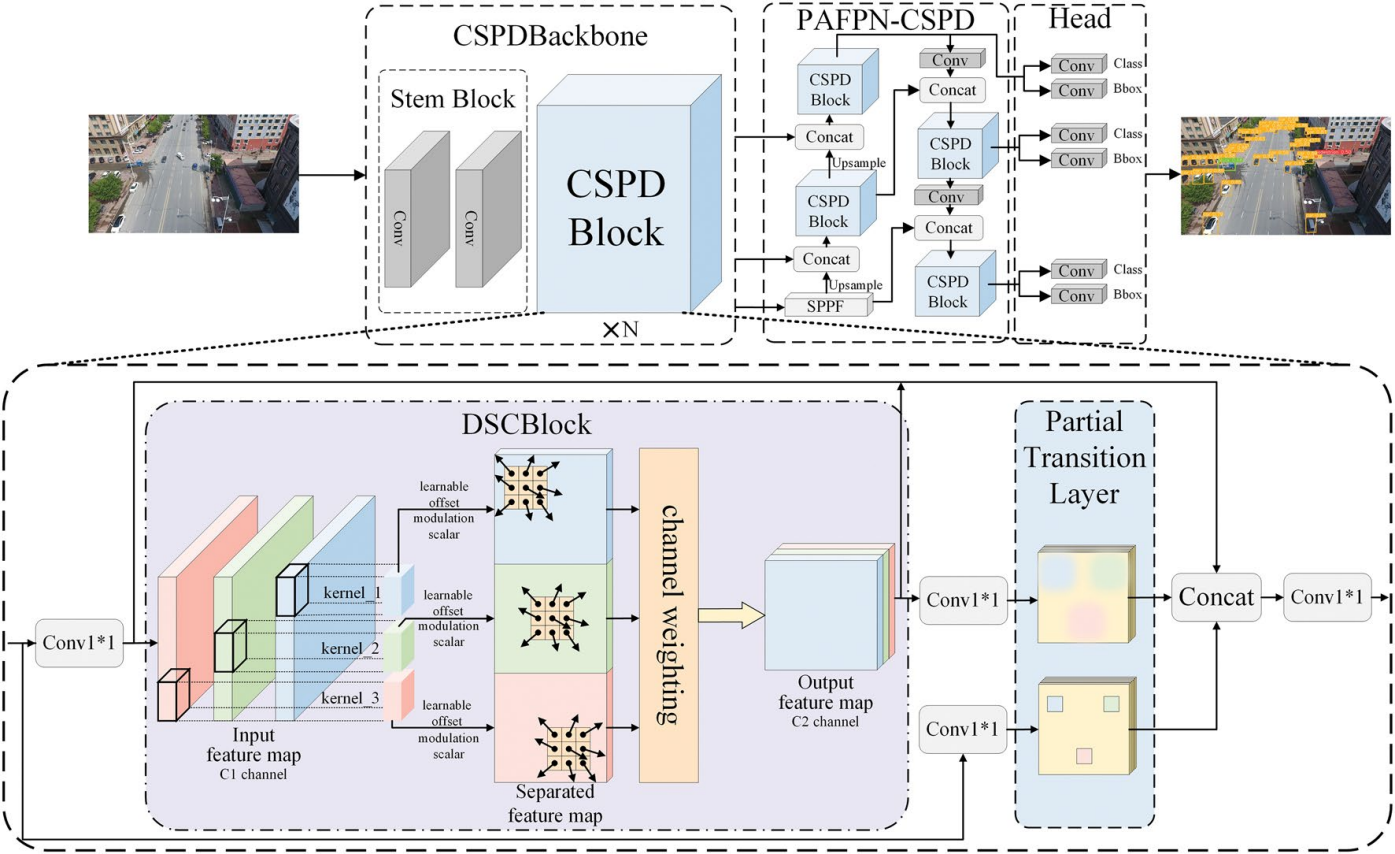
Overall architecture of CSPDNet. It is capable of reducing the parameters while maintaining accuracy and achieves efficient object detection.

To address these limitations, the Cross-Stage Partial Deformable Network (CSPDNet) design, proposed in this paper, makes the convolutional computations more efficient. The proposed modules enhance the model’s feature representation capabilities, aiming to maintain sufficient accuracy even after the application of lightweight modifications. Specifically, the primary contributions of this work are as follows:

1. To address the accuracy degradation caused by lightweight convolution operations, this paper proposes a Deformable Separable Convolution Block (DSCBlock) with adaptive cross-channel feature fusion and enhanced deformation modeling capabilities. DSCBlock focuses on image regions adaptively and establishes cross-channel features to obtain richer feature information.
2. A channel weighting module that integrates three-dimensional (3D) feature information is proposed. This module, built on efficient adaptive extensive feature extraction in two dimensions, replaces expensive pointwise (1 $\times$ 1) convolutions by applying weighting operations to the third dimension channel. It filters out more effective features and reduces the negative impact of enhanced extensive deformation modeling capability.
3. To enhance the efficiency of feature extraction in the model, this paper restructures the gradient paths and further develops an efficient computational component, Cross-Stage Partial Deformable Blocks (CSPDBlock). By controlling the longest and shortest gradient paths, CSPDBlock better balances the speed and accuracy of detection.
4. Based on the proposed modules, a new, efficient, lightweight CNN model CSPDNet is constructed. The model aims to meet the requirements of deploying networks on drones to perform target detection tasks while minimizing the performance loss caused by model reduction. Additionally, Fig. 1 illustrates the overall architecture of the proposed CSPDNet.

To sum up, the rest structure of this paper is as follows: Section "Related work" provides a review of related work regarding lightweight CNN modeling. Section Methodology introduces the pivotal features of DSCBlock along with the comprehensive structure of CSPDNet, built upon CSPDBlock. In Section "Experiments", extensive experiments are conducted, comparing them with the latest results to verify the efficacy of DSCBlock and CSPDNet. Finally, Section "Conclusion" summarize this work and outlines future research directions.

## Related work

### Lightweight convolutional module

To enable the use of network model in embedded devices, several lightweight convolutional components have emerged for CNN design. These components provide cost-effective alternatives to traditional convolutional operations. Concerning the lightweight networks architecture, MobileNetv1^13^ played a pivotal role in its inception. It primarily applied a lightweight Depth-wise Separable Convolution (DSConv) module, disassembling a full convolutional operator into two autonomous decomposition operations where the initial layer executes lightweight filtering for each input channel through Depth-Wise Convolution (DWConv). As for the second layer, it is enriched with feature information using Point-Wise Convolution (PWConv) to perform linear combinations among channels. Consequently, DSConv efficiently reduces computation load and parameters, therefore serving as the fundamental design component in subsequent lightweight network architectures.

While achieving lightweight objectives, it comes at the expense of performance. Following this trend, MobileNetv2^28^ conserves simplicity from the MobileNetv1^13^ structure and introduces IRBs to alleviate feature corruption and improve representation capabilities. The IRB paradigm serves as a fundamental module adopted by several lightweight models with diverse network architectures. Moreover, MobileNetv3^22^ enhances upon MobileNetv2 by refining the excitation module and incorporating Automatic Machine Learning (AutoML). Finally, MobileNeXt^38^ introduces hourglass blocks to mitigate information loss by flipping the structure of the inverted residue block.

As PWConv requires a significant amount of computational resources during the establishment of feature interaction, ChannelNets^39^ introduces the concept of channel-wise convolution. This approach establishes connections in the input and output dimensions, avoiding complete connectivity. It associates input and output channels through a convolutional slide, promoting enhanced information exchange between channels and the output. While each of these approaches exhibits its unique characteristics, the fundamental concept of lightweight CNN networks remains intricately linked to deep convolutional operations.

In the design of lightweight convolutional modules, the reliance on repetitive feature mapping has proven high effectiveness. However, current methods primarily emphasize cost-effective operations for lightweight enhancements, often overlooking issues of weak convolutional generalization, instability, and excessive computation. For instance, CEModule, employs a group convolutional strategy to reduce FLoating-point OPerations (FLOPs) generated during training^40^. Furthermore, it introduces a dynamic adaptation algorithm to strengthen the generalization capacity of lightweight CNN models utilizing CEModule.

### Efficient object detection network

A network constructed by lightweight convolutional component design can effectively reduce the amount of computation and model parameters; however, it will result in a certain accuracy loss. An efficient network architecture design can achieve a balance between the parametric quantity and accuracy performance. Typically, lightweight module design tends to sacrifice accuracy, yet this can be mitigated by amalgamating network architecture and accuracy-enhancing methodologies to achieve a parameter reduction without accuracy loss.

Furthermore, DenseNet^36^ provided significant inspiration for lightweight structure design. Developed upon the dense connection concept of DenseNet, a series of structural optimizations led to propose a network structure suitable for mobile devices. Added to that, by incorporating SSD^41^, the PeleeNet^42^ for object detection was introduced. CSPNet^34^ introduced the concept of Crossstage Partial Network (CSPNet) from a network structure perspective where the main goal was to achieve more abundant gradient combinations while reducing the computational effort. Therefore, CSPNet made the computational bottlenecks of PeleeNet cut into its half.

The efficient structure of Crossstage Partial Network^34^ (CSPNet) has gathered favor among several popular object detection networks. For instance, CSPDarknet53^34^ was first introduced by YOLOv4^6^ as its backbone, leading to the development of an efficient and powerful object detection model. Subsequent iterations, such as the backbone design in YOLOv5^7^, also adopted CSPDarknet53. Inspired from the previous work, PP-YOLOE^8^ introduced a novel backbone network, CSPRepResNet, integrating architectures with both residual and dense connections. As for RTMDet^43^, it constructs its backbone using CSP-blocks^34^ and deeper convolutions with larger kernel sizes.

VoVNet^35^ is designed to address the inefficiency problem as it proposes an energy and computationally efficient architecture consisting of One-Shot Aggregation (OSA). Building upon VoVNet, the CSPVoVNet^44^ architecture examines gradient paths to gather a wider array of features from various layer weights. Moreover, YOLOv7^9^ conducted an analysis regarding the gradient paths in CSPVoVNet and proposed the Extended Efficient Layer Aggregation Networks (E-ELANs) technique to design the network. This technique maintains the architecture of the transition layers while improving the network’s learning capability by altering the computational block structure through expansion, shuffling, and merging operations. Finally, the backbone network design of YOLOv8^10^ follows the core concept of E-ELAN and introduces a new module, contributing to achieve state-of-the-art results in object detection tasks.

### Efficient feature modeling

Effective feature modeling methods play a crucial role in enhancing network performance. Typically, convolutional feature sampling has been widely used and demonstrated effective for image feature extraction. However, feature modeling through sliding window with fixed-size convolution kernels cannot sample specifically according to the morphology of target features, and this sampling method is relatively inefficient. DCNv1^45^ enhances the modeling of geometric transformations by using an adaptive convolution kernel that adjusts sampling adaptively through the addition of a bias to the sampling position. DCNv2^46^ addresses the imprecise object coverage range and the problem of sample distribution extending beyond the region of interest encountered in DCNv1 by introducing a modulation mechanism. DCNv3^47^ compensates for the shortcomings of regular convolution in terms of long-range dependencies and adaptive spatial aggregation by separating the original weights into depth-wise and point-wise components. DCNv4^48^ overcomes the limitations of its predecessor, DCNv3, by removing softmax normalization in spatial aggregation to enhance its dynamism and expressive power, and optimizing memory access to minimize redundant operations for acceleration.

While lightweight convolution operations effectively reduce parameter quantity and computational load, there is some loss in feature extraction process leading to performance degradation. This paper compensates for the accuracy loss with the efficient feature modeling capability of DCNv2 to achieve a balance between speed and accuracy. Compared to standard convolution modeling operations, fewer DCNv2 can be used to establish a larger range of feature modeling, and adaptively adjusting the convolution kernel shape to more efficiently extract valid target features.

The proposed CSPDBlock enhances inter-channel feature fusion by introducing a weighted module to select feature layers that extract more valid features during the feature modeling process of DCNv2 on the separated feature layers. This paper designs lightweight models with efficient modules to meet deployment requirements with minimal accuracy loss, starting from embedded detection tasks. DCNv2 is employed to enhance feature modeling capability. Moreover, a channel weighting module is used to integrate channel information of separated feature layers. Nonetheless, it introduces a more complex sampling method compared to standard convolution, potentially consuming more memory and reducing speed. Considering the constraints of embedded devices, optimization has not yet been performed on model acceleration.

**Figure 2:**
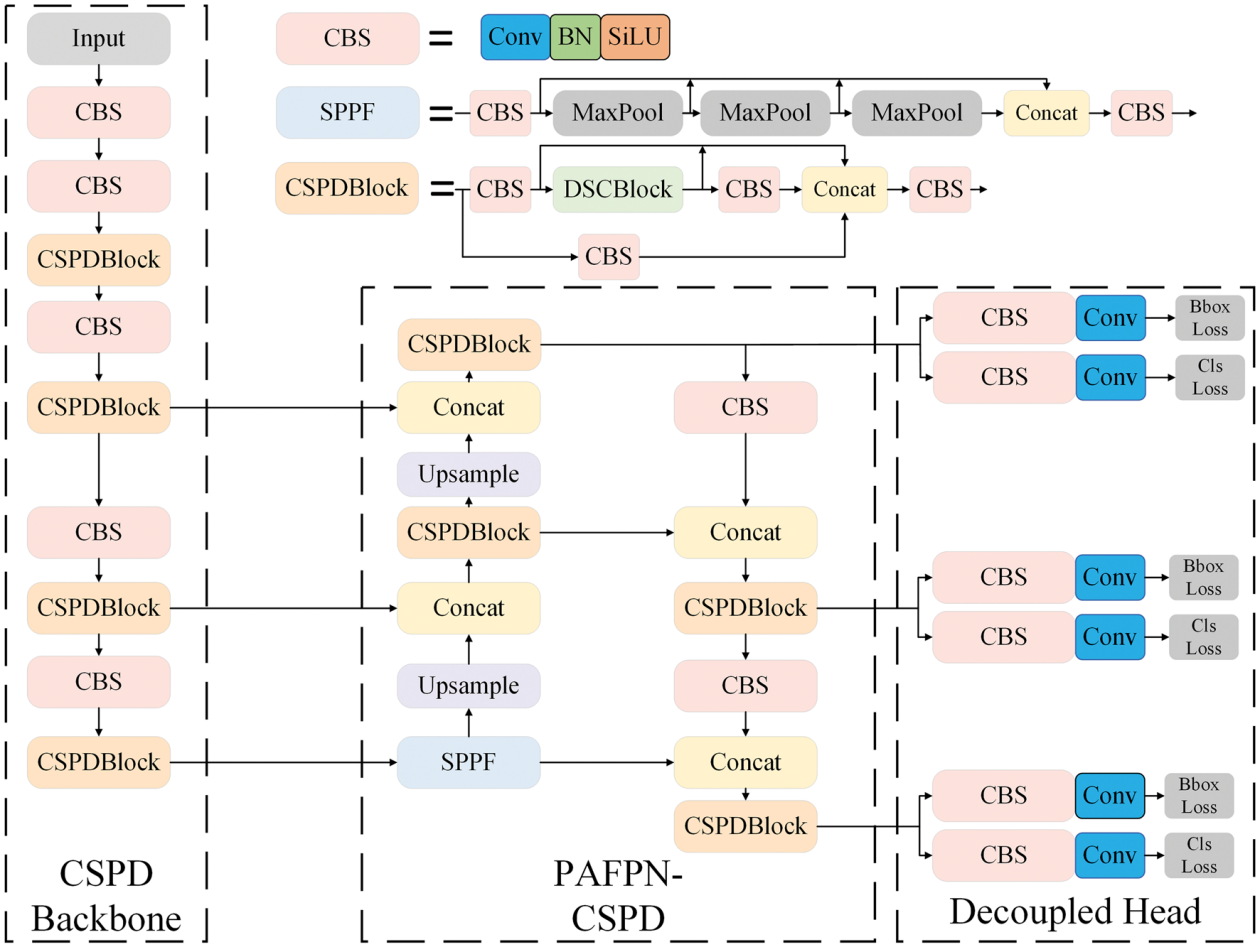
Details of the CSPDNet structure, constructing the components of the entire network.

## Methodology

This section details the design concepts and implementation specifications of CSPDNet. First, a detailed presentation of the construction of DSCBlock derived from the lightweight module design is proposed. Consequently, the structure of the gradient flow is re-optimized and DSCBlock is added serving as a basic building block to design CSPDBlock. Finally, the specific structure of the whole network is generated.

### Cross-stage partial deformable networks

Mainstream backbone network architectures, including ResNet^31^, ResNeXt^49^, and DenseNet^36^ apply linear or nonlinear combinations of feature layers as outputs. In these architectures, both residual and dense layers accumulate gradient information by merging the outputs of preceding layers. This design minimizes the length of the gradient path, enhancing the efficiency of gradient flow during back-propagation. However, it also leads to the repeated learning of redundant information.

The initial segment of CSPDNet involves two convolutions creating a Partial Transition Layer, which divides the input feature map into two segments: the first one segment stores part of the input feature data, while the other one undergoes DSCBlocking, ensuring that sufficient feature information is provided by controlling the shortest and longest gradient paths.

CSPDNet is a further development based on the design foundation of DSCBlock. The entire CSPDNet comprises a backbone network (Backbone), intermediate layers (Neck), and detection layers (Head). The structural design of the backbone network draws inspiration from CSPNet’s feature extraction architecture while leveraging DSCBlock’s efficient feature modeling capability, resulting in the CSPDBackbone. The intermediate layers (Neck) follow the design concept of PAFPN, constructed using DSCBlock. Additionally, aligned with the design of YOLOv8’s detection layers (Head), CSPDNet embraces the concept of Anchor-Free decoupled heads.

Moreover, Fig. 2 illustrates the structure and connectivity diagram of the CSPDNet network. The input image undergoes initially feature extraction through CSPDBackbone; then, it subsequently generates multi-level feature maps. These parts of information covering various stages of feature extraction, are directed into the PAFPN constructed by CSPDBlock for feature fusion, combining local and global features, and allowing the network to accommodate the multi-scale changes of the target. Finally, the decoupled heads gather feature maps from different levels to detect the targets.

Furthermore, the Backbone is constructed by alternating CBS and DSCBlock, primarily achieving the feature extraction function. In more detail, CBS denotes a standard convolution operation, comprising 2D convolution, batch normalization, and activation function. However, DSCBlock adopts the CSPNet design, comprising standard convolution, DWConv, and DConv. The Neck consist of SPPF, CBS, and Up-sample interconnected according to the PAFPN architecture, primarily achieving feature fusion. As for the Head, it consists of CBS and a 2D convolution, and generated three sets of detection results at varying resolutions, notably 20$\times$20, 40$\times$40, 80$\times$80.

In conclusion, CSPDNet effectively leverages CSPNet’s Partial Transition to curtail redundant feature learning and integrates DenseNet’s feature reuse advantages. Moreover, its efficient DSCBlock fosters multidimensional feature fusion within constrained channels, providing diverse and efficient feature modeling capabilities. Consequently, CSPDNet builds a robust backbone feature extraction network with fewer stacked modules. When compared to architectures deploying CSPNet as the backbone, CSPDNet achieves a reduced parameter count and computation while maintaining accuracy levels.

**Figure 3:**
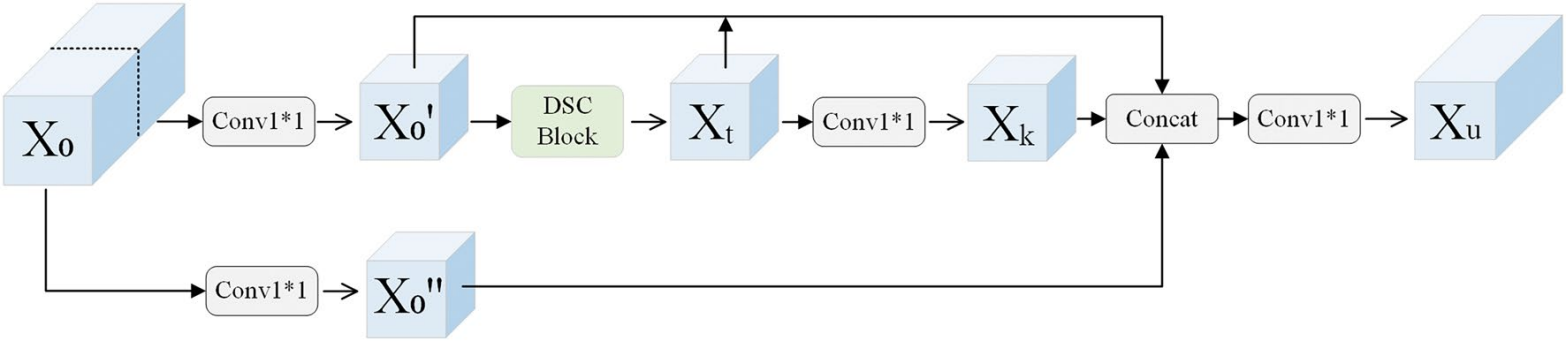
Details of the CSPDBlock structure, which has an efficient feature extraction capability and reduces the duplication of modules to form a network.

### Cross-stage partial deformable blocks

The structure of the CSPDBlock proposed in this paper is displayed in the Fig. 3. The input feature map of the CSPDBlock is initially split into two parts: the first one is utilized to retain the Partial feature from the input whereas the second part goes through the DSCBlock and enriches feature information by controlling the shortest and longest gradient paths. Moreover, within the first part, the input feature map is split into two portions using $x_0$=[$x_0'$,$x_0''$]. The $x_0''$ directly links to the end whereas, the $x_0'$ passes through the densely connected DSCBlock and the convolutional block. In addition, the second part consists of the following procedure: $x_0''$ first undergoes the DSCBlock to the output $x_t$, followed by a convolution to obtain $x_k$. Then, the output $x_k$ from the second part is connected to $x_0'$, followed by a convolutional transition layer, eventually producing the output $x_u$. The equations of the feed-forward pass and weight updating of CSPDBlock are illustrated in Eqs. 1 and 2, respectively:

1 $$\begin{aligned} \begin{aligned} {} &x_t=W_t *x_0''\\&x_k=W_k *x_t\\&x_u=W_u *[x_0',x_k,x_t,x_0''] \end{aligned} \end{aligned}$$

where $*$ represents the convolution operator, and the vector $[x_0',x_k,x_t,x_0'']$ reveals to concatenate $x_0',x_k,x_t,x_0''$, and $W_i$ representing the weights of the *i* block.

2 $$\begin{aligned} \begin{aligned} {} &W_t'=f_t(W_t,{g_0''})\\&W_k'=f_k(W_k,{g_t''})\\&W_u'=f_u(W_u,{g_0',g_k,g_t,g_0''}) \end{aligned} \end{aligned}$$

where $f_i$ denotes the function of weight updating of *i* block, and $g_i$ represents the gradient propagated to the $i^{th}$ block.

### Deformable separate convolutional blocks

DSConv^13^, a quintessential lightweight convolutional module, initially applies DWConv Filters to each input channel, then utilizes PWConv to interrelate its feature channels. Moreover, PWConv establishes relationships among feature channels. This decomposition convolutional approach significantly reduces computational load as well as the model size compared to the regular convolutional operations sequence. Nevertheless, PWConv solely facilitates the relationship between feature channels, ignoring the 3D feature information of the feature map. Moreover, in the literature^50^, 1$\times$1 convolution has been proven to still consume a significant amount of computational resources. Therefore, directly using DSConv will limit detection speed and accuracy.

To overcome this challenge, the deformable convolution (DConv)^46^ has been proven for its efficient feature modeling capability. We adaptively sample the effective receptive field for each separated channel, establishing multi-dimensional feature correlations to enrich feature information. Additionally, by modulating the amplitude of input features at different spatial positions within each feature channel, this module can more efficiently extract useful feature information.

Due to the different key information that adaptive sampling can capture on each channel, directly integrating the feature information extracted by channels may lead to conflicts and result in a decline in network performance. Therefore, a channel weighting module is used to learn the impact of the sampling results on performance for each channel, enabling the filtering of more interesting feature maps. Additionally, this module enhances the information richness contained in the weights by calculating weights for parallel multi-channel mappings.

**Figure 4:**
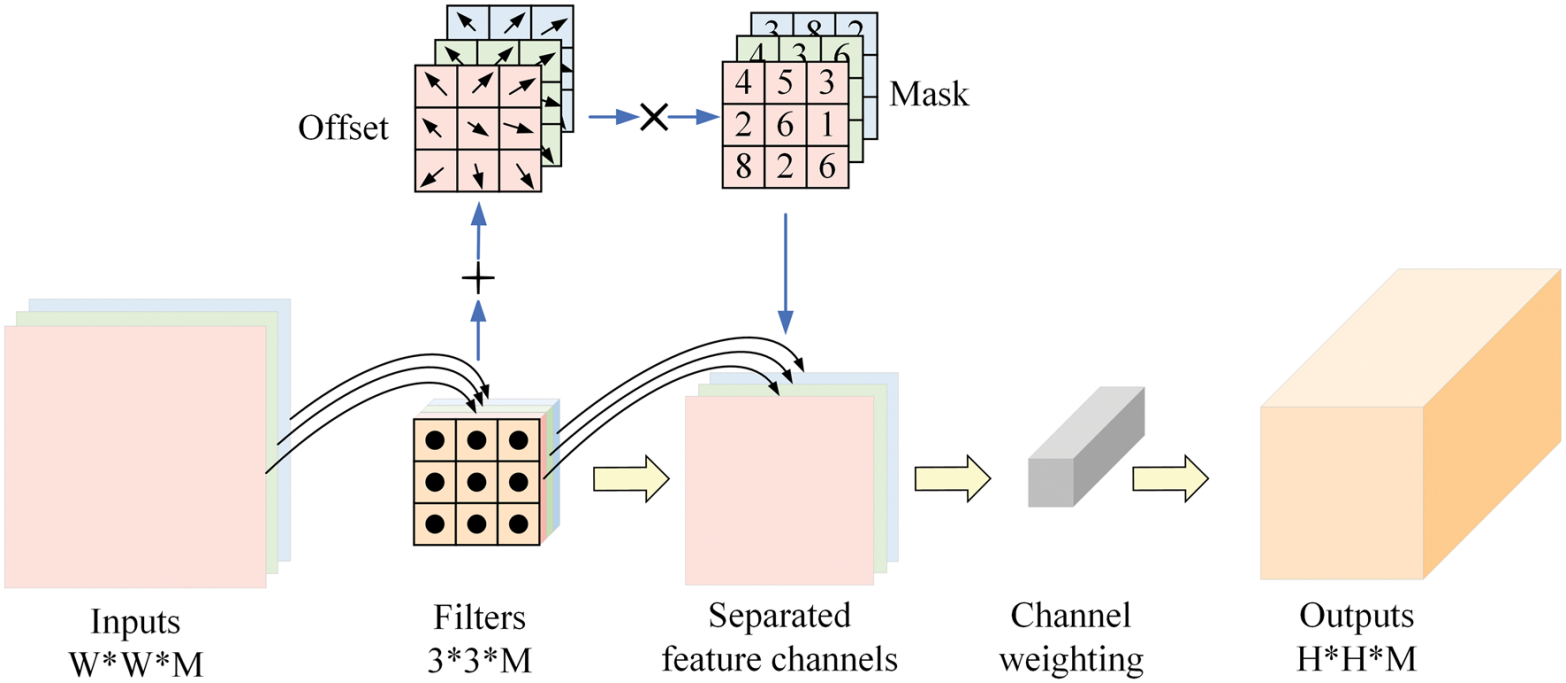
Details of the DSCBlock structure, which adaptively samples the feature map while separating channels, improving the extraction of effective features.

The structure of DSCBlock, illustrated in Fig. 4. Initially, the feature map *F* of $W \times W \times M$ dimension and is separated into feature channels using depth convolution *K*, resulting in a combined set of segregated feature channels, denoted as *S*. Subsequently, a deformable weighted convolutional operator and channel weighting is used to establish multidimensional interaction between channels and within the feature map, resulting in a feature map *G* having a dimension of $H \times H \times M$ where *W* denotes the spatial dimensions (width and height) of the input feature map, *M* represents the input channel count (depth), *H* stands for the output spatial dimensions (width and height), and *M* indicates the output channel count (depth).

The first part, the filters, performs depth convolution *K* on each input channel (depth) of feature map *F* to generate the combined feature channels *S*, represented as follows:

3 $$\begin{aligned} S_{k,l,m}=\sum _{i,j} K_{i,j,m} \cdot F_{k+i-1,m} \end{aligned}$$

As for the second part, given a convolution kernel with *p* sampling positions, it conducts deformable weighted convolution on the combined feature features and integration of channel *S* information by channel weighting to obtain feature map *G*, represented as follows:

4 $$\begin{aligned} G(p)=\sum _{k=1}^p w_k \cdot S(p + p_k +\Delta {p_k}) \cdot \Delta {m_k} \end{aligned}$$

where$w_k$ and $p_k$ denote the weights and pre-specified offsets of the $k^{th}$ position, respectively, and $\cdot$ is the multiplication operator. Let *S*(*p*) and *G*(*p*) represent the features at position *p* in the input and output feature mapping, respectively. where $\Delta {p_k}$ and $\Delta {m_k}$ denote the learnable offset and weighted modulation scalar of the $k^{th}$ position, respectively.

**Figure 5:**
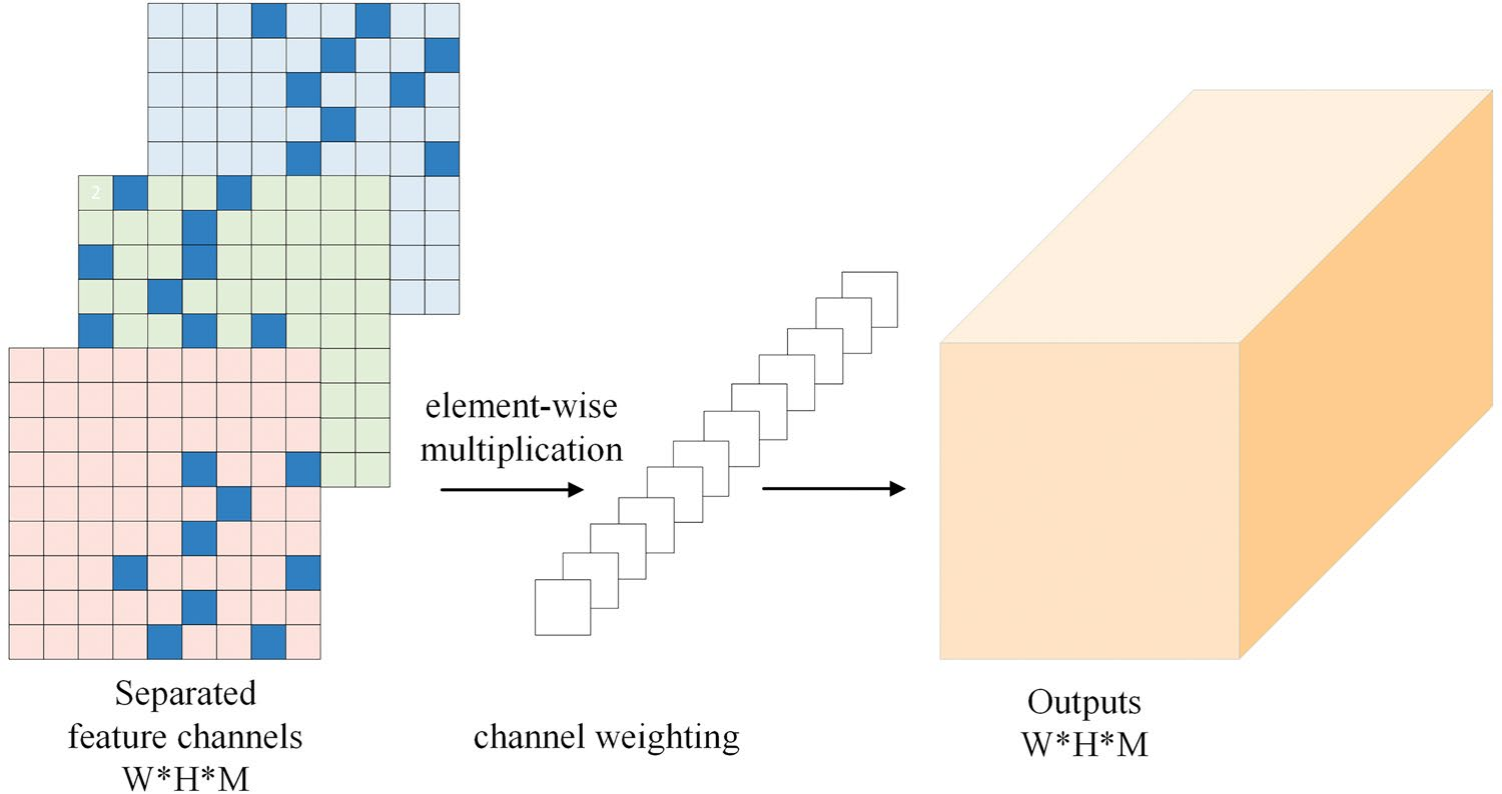
Details of the structure of the Channel Weighting Module, creating an exercise of channel dimensional information on the deformed modeled feature maps and filtering the more important information through weighting operations.

### Channel weighting module

The proposed channel weighting module involves channel-wise weighting during the two-dimensional adaptive feature extraction process, filtering out more important information, and simultaneously establishing cross-channel information correlation in the third dimension. The element-wise weighting operation across channels is expressed as follows:

5 $$\begin{aligned} Y_w=S \odot C_w \end{aligned}$$

Where *S* is a separated feature tensor with a size of $W \times H \times M$, $C_w$ is a weight mapping, and $\odot$is an element-wise multiplication operator.

We use a weighting matrix to calculate the channel dimension weights for the separated feature map. As shown in the Fig. 5, the weighted module will weigh the sampling results on each channel to obtain the output feature tensor, enhancing multi-dimensional information exchange and improving the extraction of effective features.

### Training deployment

#### Data augmentation

It is a crucial machine learning method, consisting of the generation of additional training data based on existing samples. It aims to make the augmented training data as close as possible to the real data distribution, thus enhancing detection accuracy. Moreover, data augmentation enables models to identify more robust features, effectively improving the model’s generalization capability. Added to that, the primary data augmentation methods employed in CSPDNet including: Mosaic^6^, Random Affine, Augment HSV, and Horizontal Flip.

#### Loss function

In this paper, the loss function segment of CSPDNet is similar to YOLOv8, involving two components: the positive and the negative sample allocation strategy, and loss computation. The sample allocation strategy employs in this work the Task Aligned Assigner^51^ (TAA). The decoupled head segments this function into two components, using the Binary Cross Entropy Loss for classification loss $L_{cls}$ and employing the Distribution Focal Loss^52^ for positional loss $L_{bbox}$. Therefore, the computation of the loss function and the sample allocation strategy is as follows:

1. The classification loss can be expressed as follows:

6 $$\begin{aligned} \begin{aligned} L_{cls}(y, {\hat{y}}) =&-\frac{1}{N}\sum _{i=1}^N y_i\log (\hat{y_i})\\&+ (1-y_i) \log (1-\hat{y_i}) \end{aligned} \end{aligned}$$

2. The position loss can be expressed as follows:

7 $$\begin{aligned} \begin{aligned} L_{bbox}(S_i, S_{i+1}) =&-\frac{1}{N}\sum _{i=1}^N (y_{i+1}-{\hat{y}})\log (S_i)\\&+ ({\hat{y}}-y_i) \log (S_{i+1}) \end{aligned} \end{aligned}$$

where *y* represents the true label of the sample, ${\hat{y}}$ denotes the predicted value of the model, and *N* indicates the number of samples. Moreover, $S(\cdot )$ denotes a SoftMax function.

The positive and negative sample allocation strategy can be expressed as follows:

8 $$\begin{aligned} t = s^\alpha \times u^\beta \end{aligned}$$

where *s* and *u* represent the classification score and IoU value, respectively, and $\alpha$ and $\beta$ are the weight hyperparameters. Referring to Eqs. 8, it can be seen that *t* can control the optimization of classification score as well as the IoU instantaneously to achieve the Task-Alignment process, helping the network in focusing on high-quality anchor dynamically.

## Experiments

The effectiveness of the proposed CSPDNet and its constituent module DSCBlock is validated using the UAV VisDrone^53^ object detection dataset.

### Experimental setups

#### Datasets

MS COCO^54^ has more than 200K images and 250K instances with 17 key points consisting of 80 categories. The dataset consists of a training set, a validation set, and a test set. The training set is formed of 118K images and 150K instances, the validation set contains 5K images, and the test set englobes 40K images.

VisDrone-DET2019^53^ dataset comprises 8599 images captured using UAV platforms. They serve as tasks, including urban traffic monitoring, and pedestrian detection. These images vary in location and height, offering diverse perspectives. Interpreted with over 540,000 bounding boxes across ten predefined categories, it includes training (6471), validation (548), and testing (1580) subsets. Each subset, generated from distinct locations yet resembling.

#### Performance metrics

As the number of parameters affects significantly the deployability of the embedded devices and the maintaining accuracy is paramount, the proposed evaluation focuses on the effectiveness of the lightweight module and its backbone network. Finally, the performance is evaluated based on parameter count and accuracy metrics.

Standard evaluation metrics in target detection tasks include Recall (R), Precision (P), Average Precision (AP), and mean Average Precision (mAP), and crucial indicators of detection accuracy. Among these, Intersection Over Union (IOU) measures the overlap between the Ground Truth (GT) and Proposed Result (PR) regions.

9 $$\begin{aligned} IoU = \frac{area(GT \cap PR)}{area(GT \cup PR)} \end{aligned}$$

Whether a target is detected or not is determined by setting the threshold of IOU, which is categorized into the number of True Positives (TP), False Negatives (FN), False Positives (FP), and True Negatives (TN) according to the different results of the judgment. Recall and correctness are defined as follows.

10 $$\begin{aligned} Precision= & {} \frac{TP}{TP+FP} \end{aligned}$$

11 $$\begin{aligned} Recall= & {} \frac{TP}{TP+FN} \end{aligned}$$

where TP $+$ FN denotes the number of generated bounding boxes and TP $+$ FP represent the total number of objects to be detected. Thus, P calculates the number of correctly detected objects out of the total number of objects and R calculates the number of detected objects. AP considers both accuracy and recall and is defined as follows.

12 $$\begin{aligned} AP = \int \limits _{0}^{1}P(R)dR \end{aligned}$$

It represents the average precision of detecting the same category of objects with a recall between 0 and 1. Moreover, mAP represents the average precision of all categories and is expressed as follows.

13 $$\begin{aligned} mAP = \frac{1}{D}\sum _{i=1}^{D}AP(i) \end{aligned}$$

Where D represents the number of detected object categories and i represents one of the categories. mAP metrics consider the object detection precision comprehensively. Therefore, the higher the mAP value is, the more accurate the detector will be.

**Figure 6:**
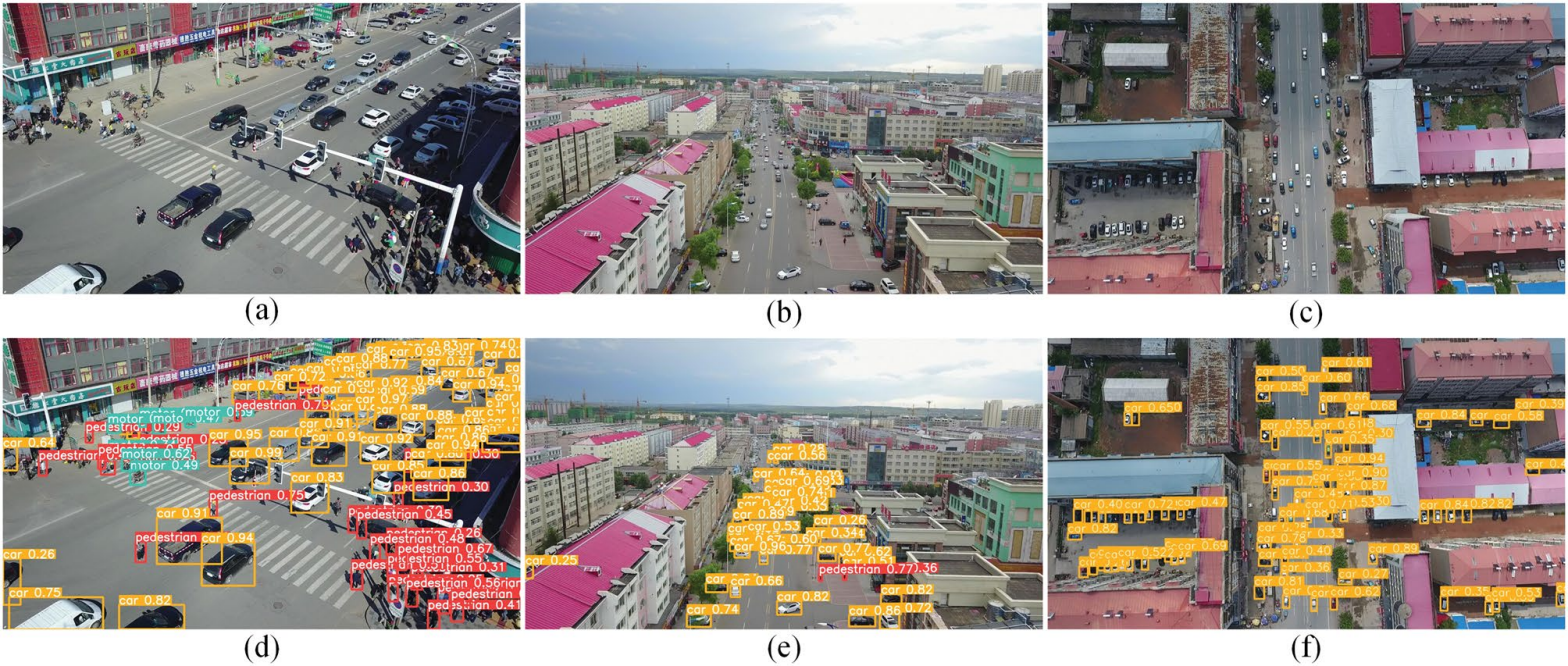
(**a**),(**b**),(**c**) show the original images to be detected, (**d**), (**e**), (**f**) show the detection results of CSPDNet.

#### Parameter settings

It represents the key parameters that must be configured. The input image size of the network is 640$\times$640 pixels. The batch size for each training is set to eight. The SGD optimization algorithm is applied in the training process, and the optimizer momentum is set to 0.937. Moreover, the Base learning rate is set to 0.01, the Base weight decay is equal to 0.0005, and the learning rate schedule is set to 0.0005. In addition, the linear rules are used where the training of the whole network is set for 300 epochs, and data enhancement is turned off in the last ten epochs to make the network converge more easily.

Experiments were conducted using Python 3.8 within the PyTorch framework, applying a single GeForce RTX 4070Ti GPU (12 GB RAM). To ensure fair comparisons, consistent training setups were employed through all experiments. The experimental comparisons, conducted using DSCBlock, involved prototypes of CSPNet and its enhanced network, CSPDNet. This latter undergoes evaluation against five other CNN models based on MMDetection’s official profile. Throughout the training process, inputs were uniformly cropped to (640 x 640). A total of 300 epochs were set for training, with a weight decay of 0.05. Due to the GPU RAM limitations, a batch size of four was chosen. The learning rate was set at 0.004.

The validation on the embedded device was realized on the Jetson Xavier NX edge computing platform. The device was configured with an Ubuntu 18.04 system, and experiments were completed using PyTorch and a single 364-core NVIDIA Volta GPU with 48 Tensor Cores.

**Figure 7:**
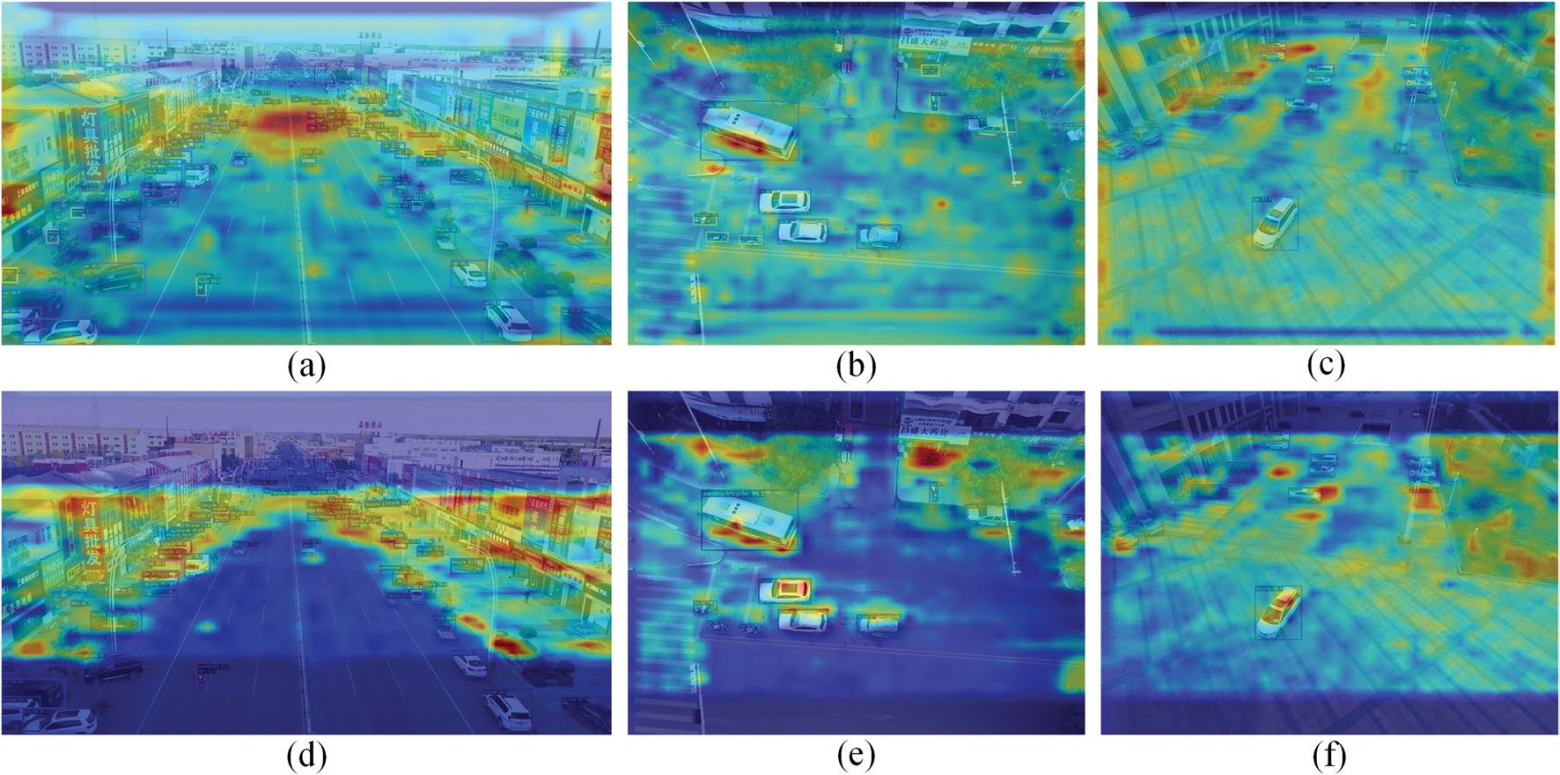
(**a**), (**b**), (**c**) show the characteristic heat maps of the baseline model, (**d**), (**e**), (**f**) show the characteristic heat maps of CSPDNet.

**Figure 8:**
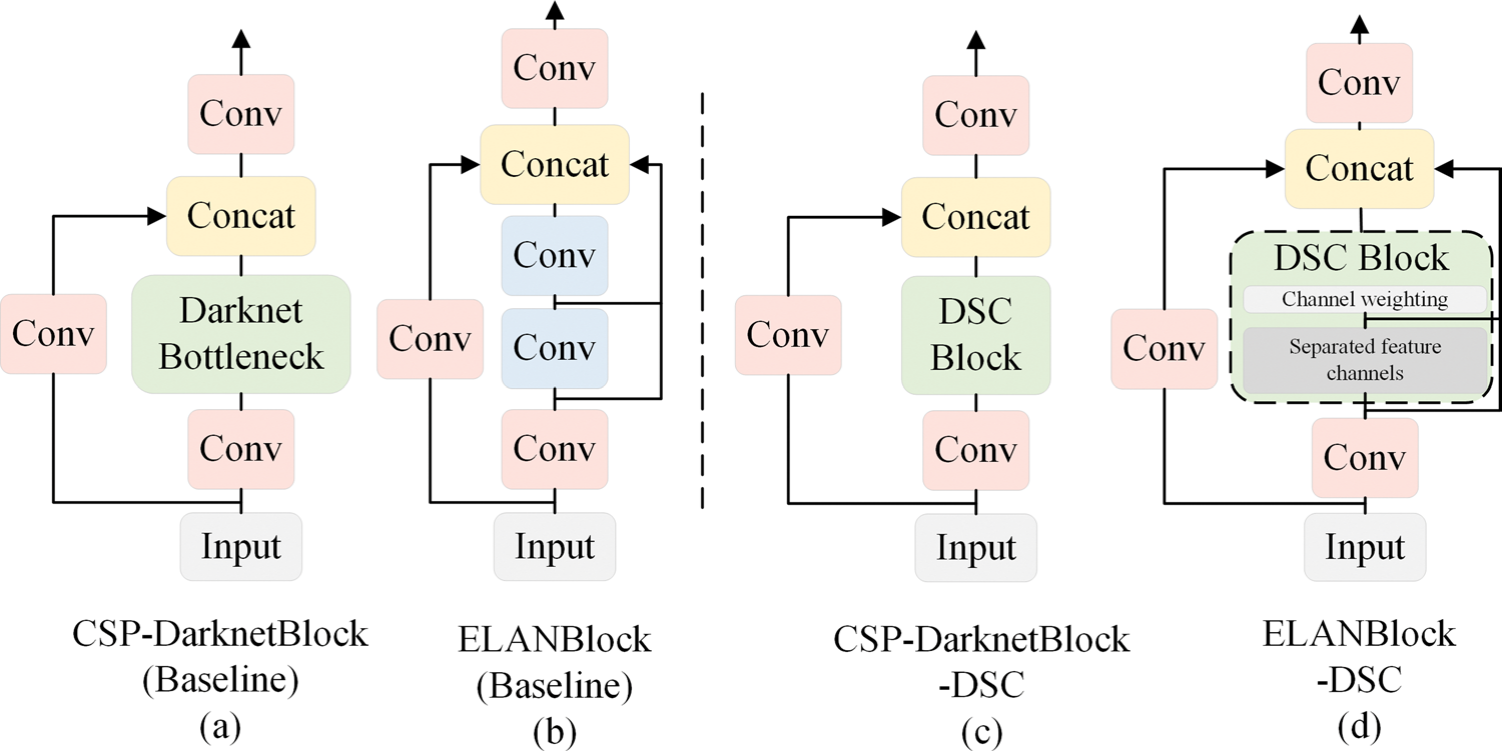
Schematic of the baseline model and the structure after replacing the module.

**Table 1:** Comparison of the parameters and accuracy of different lightweight detectors on COCO val.

Module	Parameters(MB)	Flops(G)	mAP_50:95_	FPS
MobileNetv1^13^	5.1	1.3	22.2	–
MobileNetv2^28^	4.3	0.8	22.1	–
MobileNetv3^22^	5.0	0.6	22.0	–
ShuffleNet^21^	1.9	1.5	30.5	–
NanoDet^55^	2.4	2.9	34.1	–
PP-PicoDet^56^	2.1	2.5	34.3	–
YOLOv5-n^7^	1.9	4.5	28.0	290
YOLOv6-n^57^	4.3	11.1	35.0	196
YOLOv8-n^10^(baseline)	3.2	8.7	37.3	232
CSPDNetv2-n(ours)	2.9	4.2	36.7	208
CSPDNetv4-n(ours)	2.8	4.1	36.5	216

**Table 2:** Comparison of the parameters and accuracy of different lightweight detectors on embedded devices.

Module	Parameters(MB)	Flops(G)	mAP_50_	FPS
YOLOv5-nu^7^	5.3	7.7	30.4	27.7
YOLOv7-ns^9^	11.7	13.1	35.4	17.0
YOLOv8-s^10^(baseline)	6.2	8.7	33.2	26.4
CSPDNetv2-s(ours)	5.2	7.1	32.5	24.5
CSPDNetv2-P6(ours)	8.6	7.0	34.2	24.7

### Comparing with existing lightweight networks

We select some classical and advanced lightweight detectors for comparison. The classical lightweight networks include MobileNet series^13,22,28^ and ShuffleNet^21^, whereas the advanced detectors include YOLO series^7,10,57^, NanoDet^55^, PP-PicoDet^56^, and the results refer to the original data provided in their papers and another reference^58^. Two versions of CSPDNet are provided, which use DCNv2^59^ and DCNv4^48^ to compose the CSPDBlock. Since DCNv4 improves CUDA acceleration at the GPU compared to the v2 version, the amount of computation and the number of parameters are comparable, but the inference is faster.

Referring to Table 1, we compare the performance effects of the proposed CSPDNet and existing lightweight networks on the COCO dataset. Our method achieves 36.7 mAP with less number of parameters and computational effort. It can be seen that comparing networks with comparable number of parameters, the proposed model is able to achieve higher accuracy. Comparing networks with approaches accuracy, the proposed model has fewer parameters.

The Fig. 6 shows the detection effect of CSPDNet on the VisDrone dataset, and it can be seen that the network is still able to recognize the target effectively under few parameters, which benefits from the efficient feature modeling capability.

The Fig. 7, this paper extracts the heatmaps during the feature extraction process of the CSPDNet backbone network. The depth of color in the image represents the current layer’s focus on regions in the image, with deeper colors indicating greater emphasis by the network on this area, hence, higher weights. From (a), (b), and (c) in the figure, it can be seen that the efficiency of feature modeling is enhanced by deformable convolutions, although the weights are somewhat dispersed. Subsequently, by introducing the proposed channel weighting module, the information fusion between separate feature layers can be established, resulting in (d), (e), and (f), enabling the network to focus more on extracting target feature information, further enhancing the ability to model effective features.

The Table 2 shows the validation results of the proposed CSPDNet and benchmark networks on embedded devices after being trained on the VisDrone dataset. By scaling down the number of parameters and computation, the network can be easily deployed on edge devices with limited computational and memory resources, and is able to achieve an inference speed of 25 FPS with the acceleration of hardware-supported GPUs.

### Ablation study

#### DSCBlock validation

To validate the efficacy of DSCBlock, we opted to substitute the optimized Bottlenecks within the advanced model using the foundational architecture of CSPNet. This new model maintains consistency across other components. Moreover, data results are compared on the VisDrone dataset with a Baseline model to observe alterations in both parameters count and accuracy. The network’s structure before and after the replacement is displayed in Fig. 8.

The schematic diagram illustrates the structure of the DSCBlock replacement section. In Fig. 8a, the backbone network design incorporates optimized Bottlenecks using CSPResNet^34^ serving as the foundational structure within YOLOv5^7^. Conversely, Fig. 8b showcases the component restructured within YOLOv7^9^, adopting the design concept of CSPDenseNet^34^. Subsequently, Fig. 8c,d represent the optimized structures yielding from replacing Bottleneck with DSCBlock under both foundational network types of CSPNet.

Moreover, Table 3 presents the number of parameters for both the Baseline and optimized models, along with their accuracy performance. Notably, there is a substantial reduction in the network’s parameter count after implementing DSCBlock replacement, while maintaining accuracy levels.

**Table 3:** Results of VisDrone-DET2019. Performance comparison between baseline model and after replacing DSCBlock.

Module	Parameters(MB)	Flops(G)	mAP_50_	Latency(ms)	FPS
YOLOv5s^7^	7.046	7.964	30.4	3.3	290
YOLOv7tiny^9^	6.039	13.301	35.4	5.5	179
YOLOv5s(improve)	6.127($\downarrow$13.04%)	5.178($\downarrow$34.98%)	30.3	3.7	270
YOLOv7tiny(improve)	5.333($\downarrow$11.69%)	9.810($\downarrow$26.31%)	35.5	6.6	150

#### CSPDblock validation

To verify the performance of CSPDblock at different locations in the network, disintegration experiments were conducted by adding CSPDblock to the backbone and neck layers of YOLOv8-P6^10^. Experimental comparisons were conducted on the VisDrone^53^ dataset to examine variations in model performance. The experimental results are shown in Table 4.

Experimental results indicate that adding CSPDblock at different locations effectively reduces the number of parameters and computations while maintaining accuracy within an acceptable range. Furthermore, with the incorporation of additional modules, there is a substantial reduction in both parameter and computational demands, leading to a modest enhancement in inference speed, while maintaining a minimal loss in accuracy.

**Table 4:** Results of VisDrone -DET2019. Performance comparison between baseline model and after replacing CSPDblock.

Module	Parameters(MB)	Flops(G)	mAP_50_	Latency(ms)	FPS
YOLOv8m-P6^10^	85.9	82.9	43.4	12.2	82
YOLOv8m-P6(CSPDBlock-Backbone)	72.2	59.3	42.3	10.0	100
YOLOv8m-P6(CSPDBlock-Neck)	72.6	68.0	42.5	10.1	98
YOLOv8m-P6(CSPDBlock-Backbone+Neck)	58.9	44.5	42.1	8.1	132

**Table 5:** Results of VisDrone -DET2019. Performance comparison between baseline model and after replacing CSPDNet.

Model	Backbone	Parameters of module(MB)	Parameters of backbone(MB)	Flops of module(G)	Flops of backbone(G)	mAP_50_
YOLOv5m^7^	CSPv6	20.908	12.168	24.077	15.579	36.9
PPYOLOEm^8^	RepResNet	23.428	9.041	24.965	13.302	36.6
YOLOv7m^9^	E-ELANet	36.552	13.372	105.302	67.500	48.3
RTMDetm^43^	CSPNeXt	24.669	12.279	39.093	15.714	32.8
YOLOv8m^10^	Darknet	25.903	11.856	39.566	19.341	43.7
YOLOv8s^10^	Darknet	11.129	5.082	28.457	12.346	39.5
YOLOv5m-CSPD	CSPDNet	17.869($\downarrow$14.53%)	9.129($\downarrow$24.97%)	20.246($\downarrow$15.91%)	11.748($\downarrow$24.59%)	37.0
PPYOLOEm-CSPD	CSPDNet	20.424($\downarrow$12.82%)	6.672($\downarrow$26.20%)	20.269($\downarrow$18.81%)	9.621($\downarrow$27.67%)	36.5
YOLOv7m-CSPD	CSPDNet	32.857($\downarrow$10.10%)	9.667($\downarrow$27.63%)	82.901($\downarrow$21.27%)	45.100($\downarrow$33.19%)	47.5
RTMDetm-CSPD	CSPDNet	21.518($\downarrow$12.77%)	9.129($\downarrow$25.65%)	35.126($\downarrow$10.14%)	11.748($\downarrow$25.36%)	32.8
YOLOv8m-CSPD	CSPDNet	20.495($\downarrow$20.87%)	6.489($\downarrow$45.26%)	29.369($\downarrow$25.77%)	9.229($\downarrow$52.28%)	39.1

#### CSPDBackbone Validation

Table 5 demonstrated the validation against state-of-the-art models applying CSPNet as the foundational design on the VisDrone dataset. The CSPDBackbone, a novel lightweight CNN backbone network founded on the DSCBlock, is represented. Moreover, CSPDNet predominantly integrates DSCBlock and convolution components. The YOLOv5^7^, PPYOLOE^8^, YOLOv7^9^, RTMDet^43^, and YOLOv8^10^ network models in the Table 5 are experimental comparisons performed on mmyolo^60^.

Moreover, comparative experiments are conducted utilizing advanced target detection algorithms while employing CSPNet as the basis for the feature extraction network. Without altering the network’s Neck and Head design structure, we individually replaced the backbone networks of YOLOv5^7^, PPYOLOE^8^, YOLOv7^9^, RTMDet^43^, and YOLOv8^10^. These changes were assessed through experiments on the VisDrone dataset, considering the requirements the UAV-embedded platform’s.

The backbone network CSPDBackbone proposed in this paper is mainly constructed using CSPDBlock, because the backbone network of YOLOv8 is mainly constructed by the C2f module, in which the C2f module in yolov8m multiplexes the Bottleneck. In this paper, we use CSPDBackbone to directly replace the backbone network of YOLOv8m, and the module in the corresponding position after the replacement replaces multiple multiplexed modules in YOLOv8m, which leads to a significant decrease in the number of parameters and computation in the backbone network and thus a significant decrease in the accuracy.

The performance of the yolov8-s model is supplemented in Table 5, where it can be seen that the YOLOv8m-CSPD has a comparable number of parameters in the backbone network as yolov8-s, and has a reduced computational volume, but with a very limited loss of accuracy. At this scale of computational volume, it still maintains competitive performance metrics.

For this experiment, we prioritized medium-sized models, aligning with typical volume constraints on the UAV-embedded platform. The results of the experiments, outlined in Table 5, showcase a significant reduction in the overall model size by 10–20% after replacing the Baseline model. Furthermore, the number of backbone network parameters decreased by 15–25%, with a minimal impact on accuracy.

## Conclusion

Due to the limited computational and storage resources of the UAVs mounted platform, algorithms designed to be deployed in such situations are not able to achieve excellent performance with the presence a large number of model parameters. The development of lightweight object detection algorithms can meet the needs of UAVs to perform object detection tasks, while also enhancing real-time performance and achieving better performance.

The proposed DSCBlock effectively reduces the parameter count of the advanced object detection algorithms (such as YOLOv7, YOLOv8, and RTMDet) while maintaining their superior performance. This lightweight module can be incorporated into networks, facilitating the deployment on embedded devices and edge platforms. One of its major contributions lies in multi-dimensional feature modeling of depth-wise separable convolutions and enhancing the efficiency of feature fusion, therefore enriching feature information on a larger scale. Based on these findings, CSPDNet is designed to reuse multilevel feature information in the network structure and utilizes truncated gradient flow to reduce redundant information during the reuse process. Therefore, this allows CSPDNet to use fewer parameters while reaching advanced algorithms level. Finally, experimental results indicate that our proposed DSCBlock and lightweight CSPDNet are more efficient in object detection tasks when being on parameter-limited embedded platforms compared to other outstanding already existing detection algorithms.

To sum up, these works have reduced the parameters of the detection model, but the accuracy can only be maintained within a certain range. Meanwhile, the loss of accuracy due to the characteristics of UAVs aerial images, limited by the predominance of small targets, also requires accommodation in the algorithm design. Moreover, for UAVs to perform target detection tasks, the demand for real-time performance is higher than other detection tasks.

As for the future works, based on the existing lightweight network, the network structure in the inference stage is adaptively adjusted according to the different input images to achieve the purpose of real-time detection.

## Data Availability

The datasets analysed during the current study are available at https://github.com/VisDrone/VisDrone-Dataset.
